# Copper-Mediated Leaching of LiNi_0.65_Co_0.25_Mn_0.10_O_2_ in H_3_PO_4_: Thermodynamics, Structural Evolution, and Redox Mechanism

**DOI:** 10.3390/molecules31091502

**Published:** 2026-04-30

**Authors:** Ivan Đorđević, Dragana Medić, Nataša Gajić, Maja Nujkić, Vladan Nedelkovski, Sonja Stanković, Aleksandar Cvetković

**Affiliations:** 1 Elixir Prahovo Co., Ltd., Braće Jugovića br. 2, 19330 Prahovo, Serbia; ivan.djordjevic@elixirprahovo.rs; 2 Technical Faculty in Bor, University of Belgrade, 19210 Bor, Serbia; mnujkic@tfbor.bg.ac.rs (M.N.); vnedelkovski@tfbor.bg.ac.rs (V.N.); sstankovic@tfbor.bg.ac.rs (S.S.); acvetkovic@tfbor.bg.ac.rs (A.C.); 3 Innovation Center of the Faculty of Technology and Metallurgy, Belgrade Ltd., University of Belgrade, Karnegijeva 4, 11000 Belgrade, Serbia; ngajic@tmf.bg.ac.rs

**Keywords:** lithium-ion batteries, NMC cathode, phosphoric acid leaching, copper-assisted leaching, battery recycling

## Abstract

This study investigates the leaching behavior of the LiNi_0.65_Co_0.25_Mn_0.10_O_2_ cathode material in a phosphoric acid medium, with metallic copper recycled from spent battery components serving as a reducing agent. The aim was to develop an efficient approach for the recovery of Li, Ni, Co, and Mn while providing a mechanistic understanding. Leaching experiments were performed by varying key parameters, including copper addition, acid concentration (0.2–0.8 mol·L^−1^), cathode mass (0.2–1.0 g), stirring rate (0–600 rpm), and temperature (35–80 °C). Thermodynamic analysis, supported by Pourbaix and species distribution diagrams, was used to interpret metal behavior. The results show that lithium is readily dissolved, whereas the extraction of Ni, Co, and Mn depends on the presence of copper, which enables their reduction and dissolution. Optimal conditions (0.4 mol·L^−1^ H_3_PO_4_, 0.2 g Cu, 600 rpm, 80 °C) enabled rapid extraction, exceeding 90% within 30 min, while near-complete extraction (~100%, 99%, 99%, and 97% for Li, Ni, Co, and Mn) was achieved after 60 min. Structural analysis revealed a transformation from the layered structure to spinel-like intermediates, followed by their dissolution and formation of copper phosphate phases. The proposed system represents an efficient approach for the sustainable recycling of NMC cathodes.

## 1. Introduction

Critical raw materials (CRMs) are indispensable components of modern industrial value chains, particularly in low-carbon technologies and energy storage systems, where their functional roles cannot be easily substituted [1,2]. However, the availability of CRMs is increasingly constrained by finite geological reserves and a highly uneven geographical distribution of primary resources, raising serious concerns regarding long-term supply security [2,3]. As a result, many industrialized regions are strongly dependent on imports of CRMs, exposing supply chains to economic volatility and heightened geopolitical risks [1].

In this context, the European Union (EU), facing limited access to natural resources, has established an extensive regulatory and strategic framework aimed at promoting a circular economy to reduce reliance on primary raw materials and ensure secure access to key resources. Within this framework, urban mining has emerged as a key strategy for valorizing secondary, anthropogenic sources [4]. Among these, spent lithium-ion batteries (LIBs) are particularly important due to their high lithium and transition-metal content, which is essential for advanced battery technologies [5,6,7]. It is estimated that as much as 70–82% of these resources are geographically concentrated in a limited number of countries, including China, the Democratic Republic of Congo, and Chile [8]. Furthermore, EU regulations mandate that by 2030, at least 12% of cobalt, 4% of lithium, and 4% of nickel used in batteries must originate from recycled sources, with these requirements increasing to 20%, 10%, and 12%, respectively, by 2035 [9].

In spent LIBs, valuable elements are predominantly associated with the cathode materials, which makes these components the principal focus of recycling and metal recovery strategies. Lithium-ion batteries are commonly categorized based on the nature of their cathode active materials (CAMs), such as LiCoO_2_ (LCO), LiNi_x_Co_y_Mn_1−x−y_O_2_ (NMC), LiMn_2_O_4_ (LMO), and LiFePO_4_ (LFP) [10]. Over the past decades, these materials have been systematically optimized with respect to energy density, cost, and electrochemical performance, often relying on advanced nanostructure design and additional processing steps to enhance electrochemical behavior. As a consequence of the rapid diversification and evolution of cathode chemistries, batteries currently reaching end-of-life represent a highly heterogeneous waste stream, emphasizing the need for recycling approaches that are specifically adapted to different CAM compositions. Typical cathode materials contain substantial concentrations of cobalt (5–20 wt%), nickel (5–10 wt%), manganese (10–15 wt%), and lithium (5–8 wt%), confirming their significance as high-value secondary resources [11,12,13].

Within this group, NMC-based cathodes have become particularly widespread in modern lithium-ion battery technologies, especially in applications where performance requirements must be balanced against economic constraints, such as electric vehicles. Rather than relying on a single dominant metal, NMC materials derive their functionality from the combined contribution of nickel, cobalt, and manganese. Their general composition, LiNi_x_Co_y_Mn_1−x−y_O_2_, allows systematic tuning of electrochemical behavior by adjusting the relative proportions of these transition metals, thereby influencing capacity, structural integrity, and thermal stability [14]. As a result of these adaptable properties, NMC cathodes currently represent more than half of the global market for lithium-ion battery cathode materials [15].

The operational lifetime of NMC-based batteries is typically specified at approximately 800–1000 charge–discharge cycles, corresponding to an average service period of 8–10 years. This inevitably leads to the accumulation of large quantities of spent NMC batteries entering the waste stream [15]. Importantly, the concentrations of economically valuable metals in spent NMC cathodes often exceed those found in primary mineral deposits, which strongly supports their classification as attractive urban mining resources [16]. Accordingly, effective recycling of NMC cathodes plays a dual role by contributing to the secure supply of critical raw materials while simultaneously reducing environmental and ecological risks. In this broader context, resource recovery from end-of-life LIBs aligns with the principles of green chemistry and the 3R concept (reduction, reuse, recycling), offering both environmental and economic benefits [6].

Despite these advantages, the recovery of metals from NMC-based batteries remains more technically demanding than from LCO systems, as it requires the concurrent separation and recovery of lithium, cobalt, nickel, and manganese within a single process framework [2].

In response to the increasing complexity of spent lithium-ion battery waste streams, several recycling strategies have been developed and can be broadly divided into pyrometallurgical, hydrometallurgical, and emerging alternative approaches [17,18,19,20]. Pyrometallurgical processes, which rely on high-temperature treatment to recover metallic alloys, are characterized by operational robustness and industrial maturity; however, they are associated with high energy demand, limited metal selectivity, and substantial lithium losses to slag phases. In contrast, hydrometallurgical routes are widely employed and are based on the use of acidic or alkaline aqueous solutions as lixiviants to solubilize metals from their oxide forms. The dissolved metal ions are subsequently recovered through a sequence of downstream operations, such as selective precipitation, solvent extraction, or crystallization, followed by purification steps including electrodeposition or calcination, depending on the target metal and process configuration [21]. Owing to their operation under milder conditions and their ability to enable controlled and selective recovery of individual metals, hydrometallurgical processes are particularly suitable for complex cathode chemistries such as NMC-based systems. As a result, hydrometallurgy has become the dominant approach in both academic research and industrial-scale recycling of LIBs.

In parallel with these established technologies, a range of emerging recycling methods has recently attracted attention, including bioleaching [22], electrochemical recovery [23], direct cathode regeneration [24], and solvent-based separation using deep eutectic solvents (DES) [25], particularly hydrophobic DES. These approaches offer potential advantages such as reduced chemical consumption, lower environmental impact, or direct reuse of cathode materials. Nevertheless, many of these methods remain at an early stage of technological development and are often limited by slow kinetics, low throughput, complex process control, or challenges related to scalability and economic feasibility [20,26,27,28]. Consequently, despite the growing interest in alternative recycling concepts, hydrometallurgical processing remains the most versatile and technically mature option for the efficient recovery of critical raw materials from spent NMC cathodes, providing a well-established framework for systematic kinetic and thermodynamic analysis.

Within hydrometallurgical recycling routes, metal recovery from NMC cathode materials is predominantly achieved through aqueous leaching processes. Both inorganic mineral acids and organic acids have been widely investigated as leaching agents. In most cases, efficient dissolution of transition metals requires the presence of a reducing agent to overcome kinetic limitations associated with their stable oxidation states in the cathode lattice. As a result, conventional hydrometallurgical systems are typically based on the combined action of acidic media and auxiliary reductants [2,3,10,15,29].

Among inorganic leaching systems, phosphoric acid represents a distinct medium due to its moderate acidity and its ability to interact with metal species through complexation and phosphate formation, thereby influencing both dissolution behavior and subsequent phase transformations. In the case of LiCoO_2_ cathode materials, phosphoric acid-based systems have been shown to enable efficient metal recovery, typically relying on hydrogen peroxide as a homogeneous reducing agent to facilitate the reduction of Co^3+^ and promote dissolution. While these approaches have demonstrated high extraction efficiencies and the formation of valuable phosphate products, they are intrinsically dependent on the presence of external homogeneous reducing agents, and alternative reductive strategies within phosphoric acid media have received limited attention [30,31,32,33,34].

In our previous work [35], it was demonstrated that metallic copper can act as an effective solid-state reducing agent in phosphoric acid, enabling the reductive dissolution of LiCoO_2_ without the addition of hydrogen peroxide and simultaneously affecting residue phase evolution through copper-phosphate interactions. This finding suggests that phosphoric acid-based systems can operate under fundamentally different redox regimes, in which metal-assisted reduction using recycled copper from battery current collectors replaces conventional homogeneous reducing agents. However, despite the dominant role of nickel-manganese-cobalt (NMC) cathodes in current lithium-ion battery technologies, the applicability of such reductive phosphoric acid systems to NMC materials remains largely unexplored. Existing studies involving H_3_PO_4_ either focus on selective lithium extraction, employ mixed leaching systems combining phosphoric and organic acids, or address mixed cathode waste streams without resolving the intrinsic leaching behavior of NMC as a distinct and chemically complex phase [36,37,38].

In this context, the present study systematically investigates the copper-mediated leaching of LiNi_0.65_Co_0.25_Mn_0.1_O_2_ in phosphoric acid, using recycled copper as a reducing agent. This approach extends metal-assisted reductive dissolution concepts from LiCoO_2_ systems to structurally and chemically more complex NMC cathodes. By integrating thermodynamic analysis and detailed characterization of solid residues, this work aims to elucidate the governing reaction mechanisms and phase transformation pathways in the H_3_PO_4_-Cu system, thereby contributing to the development of sustainable and chemically simplified hydrometallurgical routes for NMC battery recycling.

## 2. Results and Discussion

### 2.1. Characterization of the Cathode Material

The elemental composition of the spent cathode material was determined by ICP-OES analysis. The measured contents were 7.81 wt% Li, 29.54 wt% Ni, 20.03 wt% Co, and 21.97 wt% Mn. The presence of Ni, Co, and Mn as dominant transition metals is consistent with the composition of Li-Ni-Co-Mn (NMC) lithium-ion battery cathode materials. Variations in the relative proportions of transition metals are commonly observed for cathode materials recovered from batteries produced by different manufacturers and subjected to different service histories [11,12,39]. The remaining mass fraction is primarily attributed to oxygen, which is not quantified by ICP-OES.

**Figure 1:**
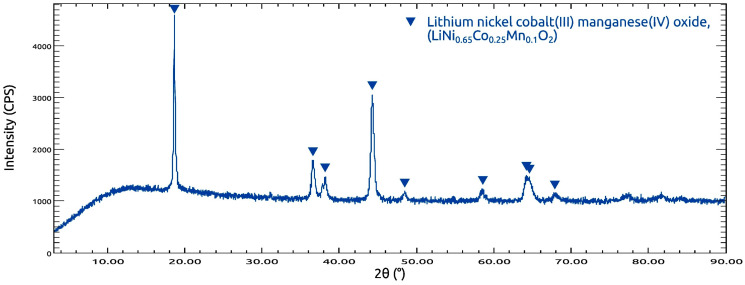
XRD pattern of the pristine LiNi_0.65_Co_0.25_Mn_0.1_O_2_ cathode material.

The XRD pattern of the investigated cathode material (Figure 1) exhibits well-defined diffraction peaks characteristic of layered lithium transition-metal oxides. The most intense reflection observed at approximately 2θ ≈ 18.7° corresponds to the (003) plane, which is a typical feature of layered Li(Ni,Co,Mn)O_2_-type cathodes and reflects the ordered stacking of lithium and transition metal layers. Additional reflections located in the regions of ~36–38°, ~44–45°, ~48–49°, ~58–59°, and ~64–66° are also characteristic of a crystalline layered structure. The overall diffraction profile is consistent with a hexagonal α-NaFeO_2_-type structure (space group $R\bar{3}m$), in which lithium and transition metal ions occupy alternating octahedral layers within a close-packed oxygen framework. Such a structural arrangement is widely reported for layered Li(Ni,Co,Mn)O_2_ cathode materials [40,41]. No additional diffraction peaks attributable to secondary crystalline phases were observed within the detection limits of the measurement, suggesting that the material predominantly consists of a layered phase.

**Figure 2:**
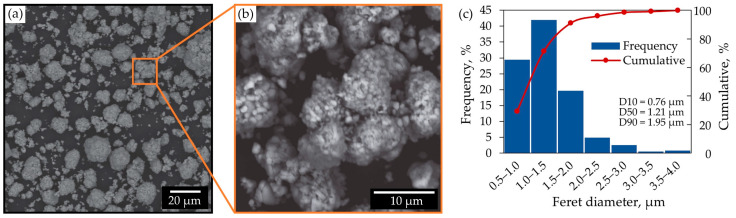
Morphology and particle size characteristics of the initial cathode material: (**a**) SEM microphotograph showing overall particle distribution and morphology; (**b**) higher-magnification view of representative agglomerates; (**c**) particle size distribution based on Feret diameter, with corresponding cumulative curve and characteristic values (D10, D50, D90).

The morphology of the initial cathode material is presented in Figure 2. The SEM microphotograph (Figure 2a) reveals that the material consists predominantly of irregularly shaped particles forming agglomerated structures. A higher-magnification image (Figure 2b) shows that these agglomerates are composed of fine primary particles, indicating a complex microstructure typical of cathode materials.

The particle size distribution, determined using ImageJ software (version 1.54g) based on the Feret diameter (Figure 2c), shows a relatively narrow distribution with D10 = 0.76 μm, D50 = 1.21 μm, and D90 = 1.95 μm, with a representative analysis example shown in Supplementary Figure S1. These results indicate that the majority of particles are within the micrometer range, which is favorable for leaching due to the increased specific surface area.

In addition, the average agglomerate size was estimated to be approximately 9 μm based on SEM microphotograph analysis (Supplementary Figure S2).

The observed agglomeration suggests that, although primary particles are fine, their clustering may influence mass transfer and dissolution kinetics during the leaching process.

**Figure 3:**
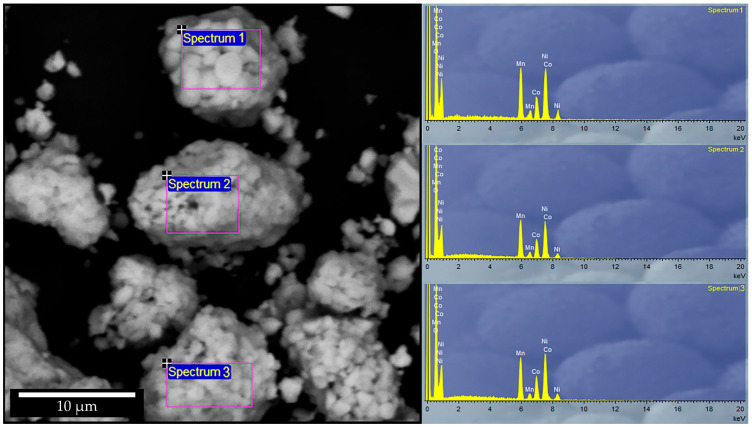
SEM microphotograph with selected EDS spectra of the initial cathode material.

SEM-EDS analysis of the initial cathode material (Figure 3) confirms a homogeneous distribution of transition metals within the agglomerates. The EDS spectra collected from different regions (Spectrum 1–3) consistently show the presence of Ni, Co, and Mn as the dominant elements, along with oxygen, indicating a typical Ni-Co-Mn oxide cathode composition.

No significant compositional variations between the analyzed regions were observed, indicating a chemically uniform distribution of elements within the agglomerates. This homogeneity is important for ensuring consistent leaching behavior of all active components.

The uniform distribution of Ni, Co, and Mn within the agglomerates indicates that all active phases are readily accessible for leaching, without pronounced elemental segregation that could hinder dissolution kinetics.

### 2.2. Thermodynamic Analysis and Speciation of the Leaching System

To provide a thermodynamic interpretation for the investigated leaching system, a set of representative reactions was defined to describe the key processes, including the dissolution of metal oxides, redox transformations, and the formation of copper phosphate. These reactions were selected to represent the dominant chemical pathways governing metal behavior under the applied conditions, namely proton-assisted dissolution, reduction by metallic copper, and subsequent precipitation of a stable metal-phosphate phase.

Li_2_O(s) + 2H^+^(a) = 2Li^+^(a) + H_2_O(l) (1)

NiO(s) + 2H^+^ = Ni^2+^+H_2_O(l) (2)

Co_3_O_4_(s) + 8H^+^ = Co^2+^ + 2Co^3+^ + 4H_2_O(l) (3)

Co_3_O_4_(s) + 8H^+^ + Cu = 3Co^2+^ + Cu^2+^ + 4H_2_O(l) (4)

MnO_2_(s) + 4H^+^ + 2e^−^ = Mn^2+^ + 2H_2_O(l) (5)

MnO_2_(s) + 4H^+^ + Cu = Mn^2+^ + 2H_2_O(l) + Cu^2+^ (6)

3Cu^2+^ + 2PO_4_^3−^ = Cu_3_(PO_4_)_2_(s) (7)

H_3_PO_4_ = H^+^(a) + H_2_PO_4_^−^(a) (8)

H_2_PO_4_^−^(a) = H^+^(a) + HPO_4_^2−^(a) (9)

HPO_4_^2−^(a) = H^+^(a) + PO_4_^3−^(a) (10)

Thermodynamic parameters (ΔH^θ^, ΔS^θ^, ΔG^θ^, and equilibrium constant, log K) were calculated for each reaction using the Reaction Equations module in HSC Chemistry over the temperature range of 0–100 °C. For clarity and relevance to experimental conditions, selected values at 0, 35, 60, 80, and 100 °C are presented in Table 1. These temperatures correspond to the key intervals used in the experimental study and enable direct comparison between thermodynamic predictions and observed leaching behavior.

**Figure 4:**
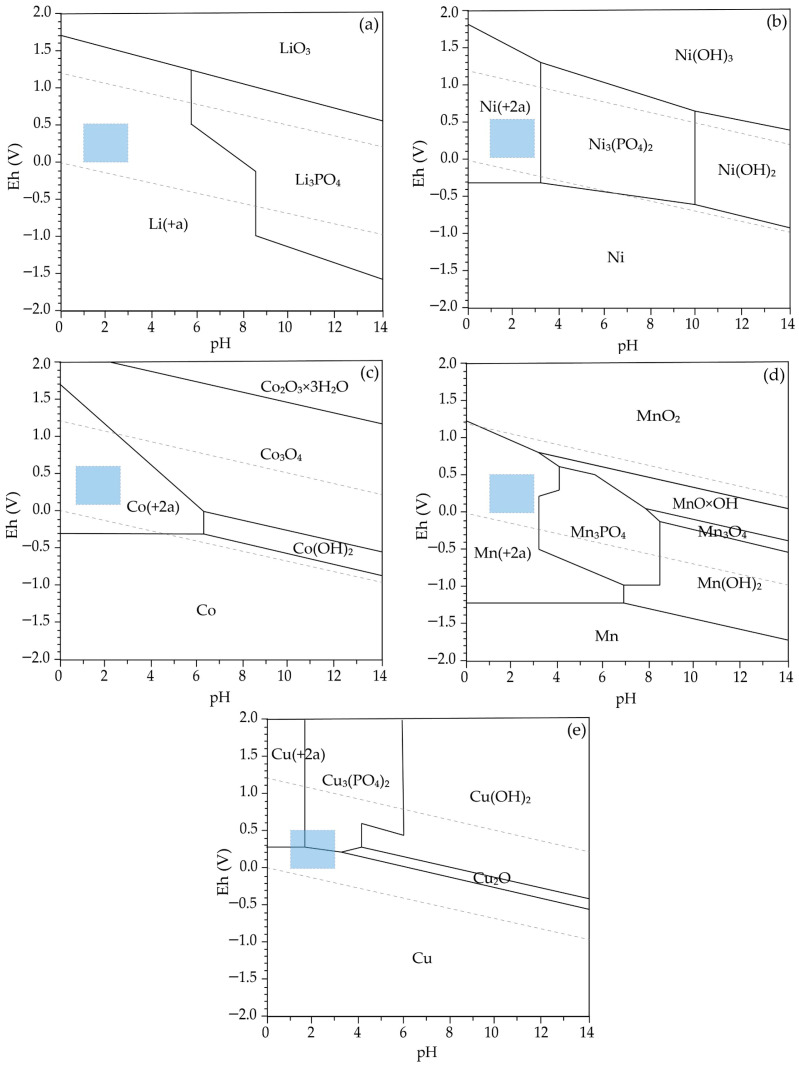
Eh-pH (Pourbaix) diagrams for (**a**) Li, (**b**) Ni, (**c**) Co, (**d**) Mn, and (**e**) Cu at 80 °C and 1 atm. Blue shaded areas indicate the experimental pH–Eh region; “(+a)” denotes aqueous species. Initial concentrations: Co (0.03 M), Ni (0.04 M), Li (0.09 M), P (0.40 M), Cu (0.06 M), and Mn (0.03 M).

**Figure 5:**
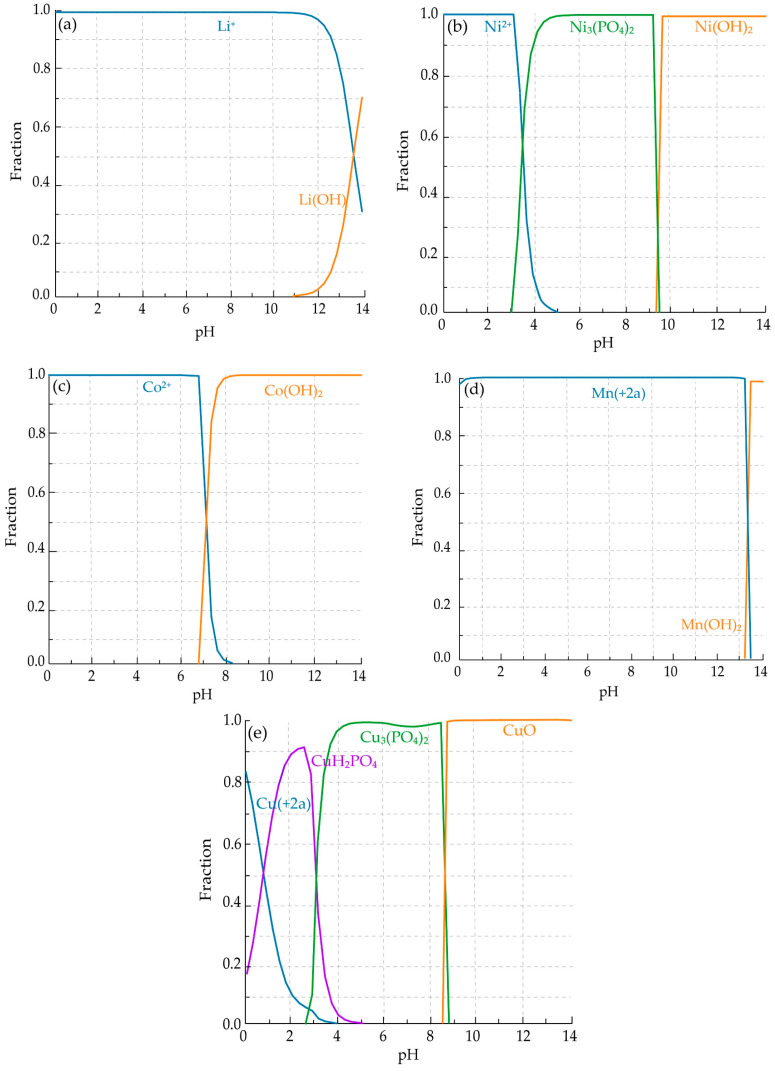
Species distribution (fraction) diagrams for (**a**) Li, (**b**) Ni, (**c**) Co, (**d**) Mn, and (**e**) Cu systems, showing the relative abundance of aqueous species and solid phases as a function of pH, calculated at 80 °C and 1 atm for input concentrations of Co (0.03 M), Ni (0.04 M), Li (0.09 M), P (0.40 M), Cu (0.06 M), and Mn (0.03 M).

The calculated thermodynamic parameters (ΔH^θ^, ΔS^θ^, ΔG^θ^, and log K) for the selected reactions clearly indicate that the dissolution of lithium and nickel oxides in acidic media is thermodynamically favorable over the entire investigated temperature range. The strongly negative ΔG^θ^ values for reaction (1) confirm that Li_2_O readily dissolves to form Li^+^, which is consistent with the Pourbaix diagram (Figure 4a) where lithium exists exclusively as a dissolved ionic form across the relevant pH range. This behavior is further supported by the species distribution diagram (Figure 5a), showing complete dominance of Li^+^ without formation of secondary phases.

Similarly, the negative ΔG^θ^ values for reaction (2) indicate spontaneous dissolution of NiO. The corresponding Pourbaix diagram (Figure 4b) confirms that Ni^2+^ is the stable species under acidic conditions, while the species distribution diagram (Figure 5b) shows that nickel remains predominantly in solution, with only minor formation of solid phases at higher pH values.

In contrast, the dissolution of cobalt strongly depends on the redox conditions of the system. Reaction (3), describing the dissolution of Co_3_O_4_ without a reducing agent, exhibits positive ΔG^θ^ values, indicating that this process is not thermodynamically favored. However, in the presence of metallic copper, reaction (4) becomes highly favorable, as reflected by the strongly negative ΔG^θ^ values and high log K. This transition is consistent with the Pourbaix diagram (Figure 4c), which shows that Co^3+^-containing phases are stable under oxidizing conditions, while Co^2+^ is stabilized only under reducing conditions. The corresponding species distribution diagram (Figure 5c) confirms that cobalt is present as Co^2+^ in the acidic region relevant to the leaching process.

A similar behavior is observed for manganese. The reduction of MnO_2_ to Mn^2+^, represented by reactions (5) and (6), is thermodynamically favorable, as indicated by negative ΔG^θ^ values. However, the Pourbaix diagram (Figure 4d) indicates that Mn^2+^ is stable only under sufficiently reducing conditions, whereas Mn(IV) oxides remain stable in oxidizing environments. The species distribution diagram (Figure 5d) further confirms that Mn^2+^ dominates only within the acidic and reducing region, explaining the experimentally observed dependence of manganese leaching efficiency on the presence of copper.

Copper exhibits a dual role in the system. Initially, it acts as a reducing agent, facilitating the dissolution of cobalt and manganese while being oxidized to Cu^2+^.

The Pourbaix diagram (Figure 4e) indicates the stability of Cu^2+^ under acidic conditions; however, it does not account for the presence of phosphate species. In contrast, the species distribution diagram (Figure 5e) demonstrates that Cu^2+^ is progressively converted into phosphate complexes and ultimately precipitates as Cu_3_(PO_4_)_2_. This behavior is consistent with the strongly negative ΔG values of reaction (7), confirming the thermodynamic favorability of copper phosphate formation. These results indicate that, although Cu^2+^ is formed as an intermediate during copper oxidation, it is thermodynamically unstable in the presence of phosphate ions and is rapidly removed from the solution through precipitation, leading to the formation of solid copper phosphate phases under the investigated conditions.

**Figure 6:**
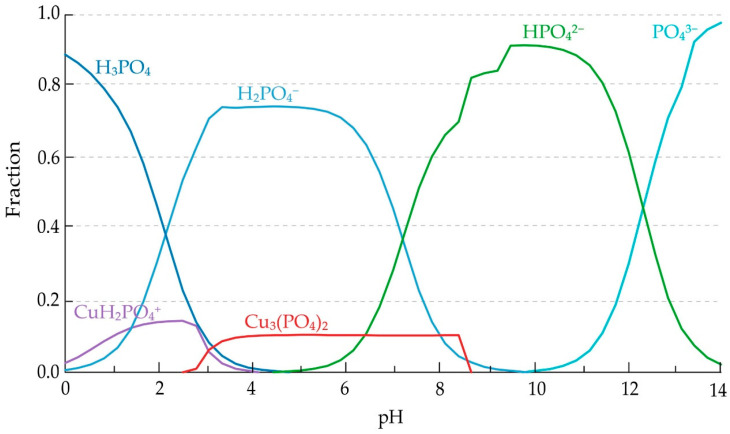
Species distribution of phosphoric acid indicating dominant phosphate species as a function of pH under leaching conditions (80 °C, 1 atm).

The dissociation equilibria of phosphoric acid, described by reactions (8)–(10), play a crucial role in controlling the availability of phosphate species. The corresponding species distribution diagram (Figure 6) shows that H_3_PO_4_ and H_2_PO_4_^−^ dominate under strongly acidic conditions, while HPO_4_^2−^ and PO_4_^3−^ become significant at higher pH values. This speciation governs the formation of copper phosphate species, facilitating the transition from dissolved Cu^2+^ to solid phosphate phases during leaching.

It should be noted that the thermodynamic analysis is based on idealized, well-defined phases available in the database, such as Cu_3_(PO_4_)_2_. However, under real aqueous conditions, the formation of copper phosphate phases is strongly influenced by pH, phosphate speciation, and hydration effects. As a result, the precipitated phases may differ in structure and composition from the idealized phases used in thermodynamic modeling, often appearing as hydrated or protonated forms. Therefore, the thermodynamic calculations should be interpreted as indicating a general tendency for copper phosphate formation, while the exact phase composition depends on solution chemistry and crystallization conditions.

The combined interpretation of Pourbaix diagrams (Figure 4a–e) and species distribution diagrams (Figure 5a–e) provides a comprehensive understanding of the system. While Pourbaix diagrams define the thermodynamic stability domains as a function of pH and redox potential, species distribution diagrams quantify the relative abundance of individual species and reveal transformation pathways that are not visible in Eh-pH diagrams.

Overall, the thermodynamic analysis indicates that acidic and reducing conditions represent the optimal region for leaching. This region, highlighted in the Pourbaix diagrams (Figure 4), corresponds to an approximate pH range of 1–3 and Eh values between 0 and +0.5 V, where Li^+^, Ni^2+^, Co^2+^, and Mn^2+^ are stable in solution, while copper undergoes transformation from a metallic reductant to dissolved Cu^2+^ and finally to stable phosphate phases (Figure 5e). The consistency between ΔG calculations, equilibrium constants, Pourbaix diagrams, and species distribution diagrams reflects their common thermodynamic basis and supports the internal coherence of the model. Importantly, these predictions are in good agreement with the experimentally observed leaching behavior.

The speciation of phosphoric acid, presented in Figure 6, provides a detailed insight into the distribution of phosphate species as a function of pH under the investigated conditions. It can be observed that H_3_PO_4_ and H_2_PO_4_^−^ dominate in the strongly acidic region relevant to the leaching process, while HPO_4_^2−^ and PO_4_^3−^ become significant only at higher pH values. The predominance of H_2_PO_4_^−^ in the leaching-relevant domain indicates limited availability of free PO_4_^3−^ ions, suggesting that phosphate precipitation processes are strongly governed by local chemical conditions and metal-ligand interactions. This behavior directly influences the formation of copper phosphate and explains the progressive transformation of Cu^2+^ species observed during leaching.

The thermodynamic analysis presented above provides a consistent thermodynamic basis for understanding the behavior of lithium, nickel, cobalt, and manganese in the H_3_PO_4_-Cu system, indicating that acidic and reducing conditions favor the dissolution of all investigated metals while promoting the transformation of copper into stable phosphate phases.

To verify these predictions and to determine the optimal operating conditions, a series of leaching experiments was conducted under controlled conditions. The following subsections examine the influence of key process parameters, including the presence of a reducing agent (copper), phosphoric acid concentration, solid-to-liquid ratio, stirring rate, and temperature, on the leaching efficiency of the investigated metals.

### 2.3. Optimal Leaching of Waste Cathode Material

#### 2.3.1. Effect of Copper Powder on the Leaching Efficiencies of Li, Ni, Co, and Mn

**Figure 7:**
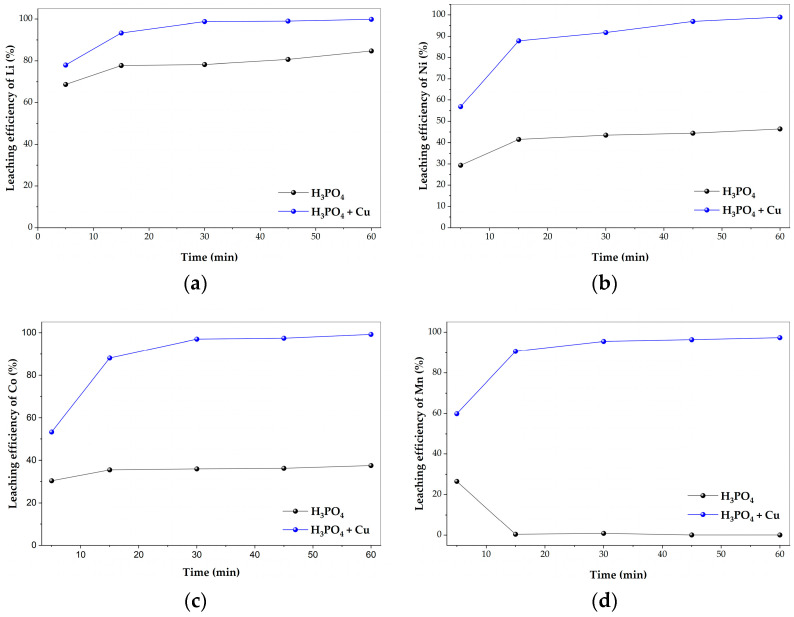
Time-dependent leaching efficiencies of metals at 80 °C in the presence and absence of 0.2 g Cu: (**a**) Li, (**b**) Ni, (**c**) Co, and (**d**) Mn.

To evaluate the influence of metallic copper as a reducing agent, comparative leaching experiments were conducted in 50 mL of 0.4 mol·L^−1^ H_3_PO_4_ at 80 °C using 0.4 g of cathode material and a stirring speed of 600 rpm, with and without the addition of 0.2 g Cu. The obtained results are presented in Figure 7.

Lithium exhibited substantial dissolution even in the absence of copper, reaching approximately 85% after 60 min. The addition of copper significantly enhanced lithium extraction, achieving nearly complete dissolution (~99.8%) under the same conditions. In the presence of copper, lithium extraction increased rapidly during the initial stage, reaching approximately 98.7% within 30 min, after which the system approached equilibrium. Although lithium is present in the +1 oxidation state and does not require reductive transformation before dissolution, the pronounced effect of copper suggests a possible indirect mechanism related to structural destabilization of the cathode material induced by enhanced transition metal dissolution.

In contrast, a pronounced effect of copper was observed for transition metals. In the absence of copper, cobalt and nickel extraction remained limited, reaching approximately 38% and 47%, respectively, after 60 min. However, in the presence of copper powder, both metals dissolved rapidly, exceeding ~88% within the first 15 min and approaching nearly complete extraction (~99%) after 45–60 min.

The most pronounced effect was observed for manganese. Without copper, manganese dissolution remained negligible throughout the experiment, staying below 1% after 60 min. Interestingly, a transient increase in manganese concentration (~27%) was observed at 5 min, followed by a sharp decrease at longer times. This behavior suggests that manganese may initially dissolve but could subsequently undergo reprecipitation or reoxidation into less soluble forms, such as Mn(III/IV) oxides or phosphate-containing phases. In contrast, in the presence of copper, manganese appears to be more efficiently leached, reaching approximately 97% after 60 min, with more than 90% extraction achieved within 15 min. These experimental observations are consistent with thermodynamic predictions.

The dissolution of Ni is favorable in acidic media even without a reducing agent, which explains its partial extraction. However, the incomplete recovery indicates that nickel dissolution is influenced by structural constraints and kinetic limitations rather than thermodynamic restrictions. In contrast, cobalt dissolution is not thermodynamically favored under non-reducing conditions but becomes highly favorable in the presence of copper, confirming the key role of Cu as a reducing agent. A similar trend is observed for manganese: although the reduction of MnO_2_ to Mn^2+^ is thermodynamically favorable, the absence of a reducing environment may limit its stability in solution, potentially leading to reoxidation or reprecipitation. The presence of copper ensures a continuous electron supply, stabilizing Mn^2+^ and enabling efficient leaching.

**Figure 8:**
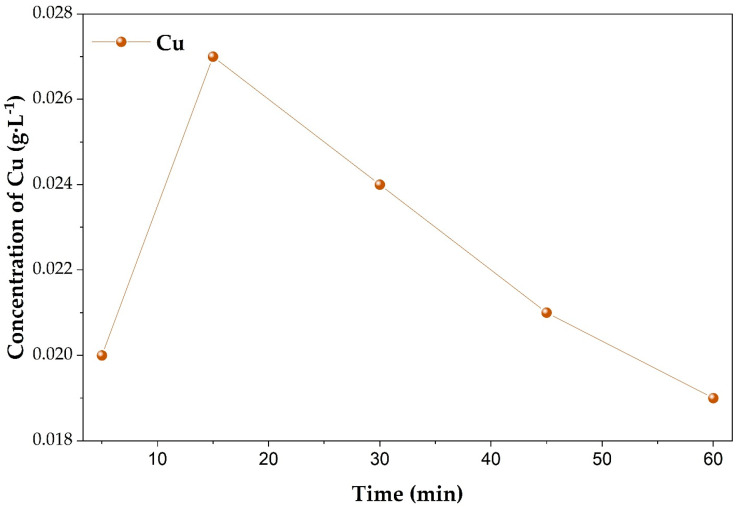
Time-dependent concentration of dissolved Cu during leaching at 80 °C.

The concentration of dissolved copper during leaching (Figure 8) increased from 0.020 g·L^−1^ after 5 min to a maximum of 0.027 g·L^−1^ at 15 min, followed by a gradual decrease to 0.019 g·L^−1^ after 60 min. This trend confirms that copper actively participates in the reaction system rather than acting as an inert solid additive. The initial increase indicates oxidation of metallic Cu to Cu^2+^, whereas the subsequent decrease suggests further redox transformations and subsequent precipitation of copper phosphate phases in the phosphoric acid medium, which is consistent with the formation of copper phosphate phases identified by XRD analysis.

The enhanced dissolution of transition metals in the presence of copper can be attributed to reductive destabilization of high-valence species within the layered NMC structure. In these materials, cobalt and manganese are partially present in higher oxidation states (Co^3+^ and Mn^4+^), which are stabilized by strong metal-oxygen bonds within the oxide lattice. This stabilization reduces the thermodynamic driving force for proton-assisted dissolution in acidic media, resulting in limited solubility under non-reducing conditions [42,43,44].

Reduction to the divalent state (Co^2+^ and Mn^2+^) weakens the metal-oxygen bonds, disrupts the layered oxide framework, and facilitates metal release into solution. The negligible dissolution of manganese in the absence of copper, together with its rapid extraction under reductive conditions, strongly supports the necessity of a reduction pathway for efficient Mn leaching. The presence of metallic copper promotes electron transfer reactions that convert Co^3+^ and Mn^4+^ into their more soluble divalent forms, thereby significantly enhancing overall leaching efficiency.

These findings indicate that copper functions as both a reductant and a redox mediator in the H_3_PO_4_ system. Under non-reductive conditions, high oxidation states remain stable and limit metal dissolution, whereas the introduction of metallic Cu shifts the redox environment toward more favorable reduction potentials, enabling efficient transition metal extraction. A similar copper-assisted effect has been reported in the leaching of LiCoO_2_ in phosphoric acid systems, where metallic Cu promotes cobalt reduction and significantly improves extraction efficiency [35]. The present results extend this concept to the more complex LiNi_0.65_Co_0.25_Mn_0.1_O_2_ system, demonstrating that the role of copper becomes even more critical for enabling manganese dissolution in multi-component cathode materials. Overall, the results confirm that reductive assistance is essential for efficient transition metal extraction in H_3_PO_4_ media. Metallic copper, therefore, represents a viable and effective alternative to conventional reducing agents and provides a simplified and effective strategy in phosphate-based hydrometallurgical systems. Based on these findings, copper powder was employed in all subsequent experiments.

#### 2.3.2. Effect of Phosphoric Acid Concentration on the Leaching Efficiencies of Li, Ni, Co, and Mn

**Figure 9:**
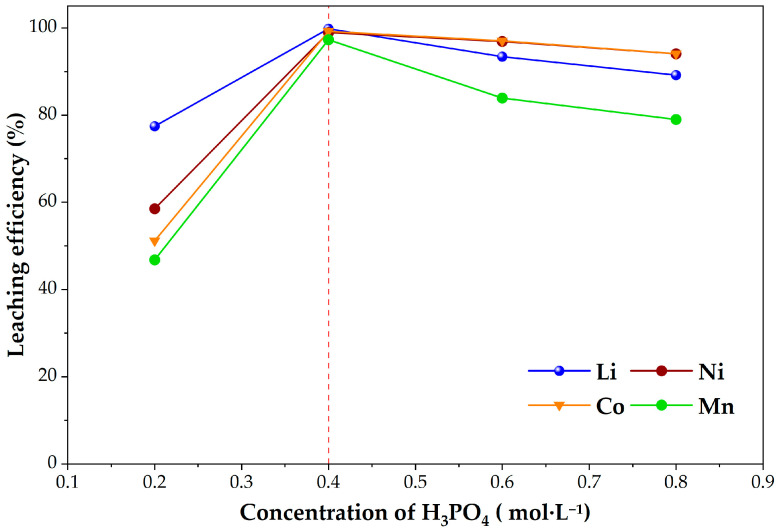
Effect of H_3_PO_4_ concentration on the leaching efficiencies of Li, Ni, Co, and Mn (0.2 g Cu, 0.4 g cathode material, 80 °C, 600 rpm, 60 min). The dashed line indicates the optimal H_3_PO_4_ concentration (0.4 mol·L^−1^).

The influence of phosphoric acid concentration on metal extraction was evaluated at 80 °C in the presence of 0.2 g Cu, maintaining a stirring speed of 600 rpm, after 60 min of leaching. The H_3_PO_4_ concentration was varied from 0.2 to 0.8 mol·L^−1^. The obtained results are presented in Figure 9.

Increasing the acid concentration from 0.2 to 0.4 mol·L^−1^ resulted in a pronounced improvement in the leaching efficiencies of all investigated metals. Lithium extraction increased from 77% at 0.2 mol·L^−1^ to 99% at 0.4 mol·L^−1^. A similar trend was observed for transition metals: nickel extraction increased from 58% to 98%, cobalt from 51% to 99%, and manganese from 46% to 97%.

However, further increase in acid concentration to 0.6 and 0.8 mol·L^−1^ did not lead to additional improvement. In the case of Ni and Co, extraction efficiencies remained high but showed a slight decrease (approximately 96–94% for Ni and 97–94% for Co). For Li and Mn, a more noticeable decline was observed, with lithium extraction decreasing to about 93% and 89% and manganese to approximately 84% and 79% at 0.6 and 0.8 mol·L^−1^, respectively.

These results indicate that 0.4 mol·L^−1^ H_3_PO_4_ provides sufficiently high extraction efficiencies for all investigated metals without the need for higher acid concentration. The absence of further improvement at elevated acid concentrations suggests that excessive proton availability does not enhance dissolution under the applied conditions. Instead, the observed decrease in leaching efficiency at higher H_3_PO_4_ concentrations can be attributed to several coupled effects. First, the increased availability of phosphate species promotes the formation of aqueous metal-phosphate complexes, which modify metal speciation and reduce the activity of free metal ions, thereby decreasing the effective driving force for dissolution [45]. Second, the higher ionic strength of more concentrated phosphoric acid solutions can modify the activity coefficients of dissolved species and alter the effective proton activity and interfacial reaction environment [46]. In addition, in the presence of metallic copper, elevated phosphate concentrations favor the formation of secondary copper phosphate phases on or near reactive surfaces. The accumulation of such phosphate-rich layers decreases the available copper surface for electron transfer and leads to partial passivation of the solid–liquid interface, thereby hindering Cu-mediated reductive dissolution of transition metals [35]. This interpretation is further supported by the XRD results obtained in this study, which confirm the progressive formation and dominance of copper phosphate phases in the solid residues. Therefore, 0.4 mol·L^−1^ H_3_PO_4_ was selected as the optimal concentration for subsequent experiments.

#### 2.3.3. Effect of Cathode Material Mass on the Leaching Efficiencies of Li, Ni, Co, and Mn

**Figure 10:**
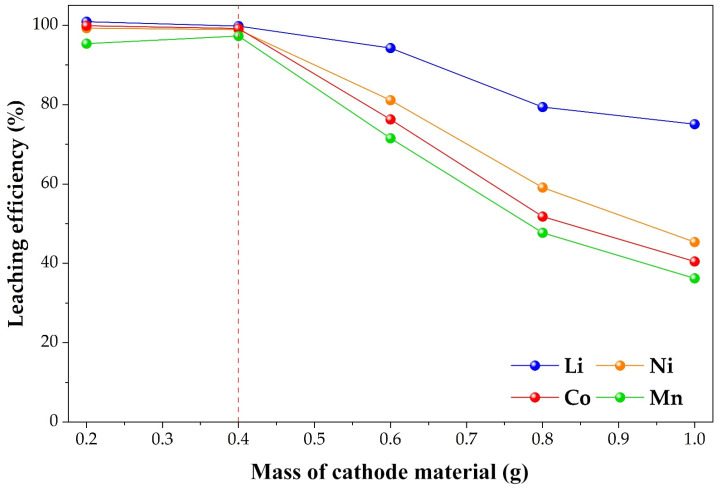
Effect of cathode material mass on the leaching efficiencies of Li, Ni, Co, and Mn (0.2 g Cu, 0.4 mol·L^−1^ H_3_PO_4_, 80 °C, 600 rpm, 60 min). The dashed line indicates the optimal cathode material mass (0.4 g).

The influence of cathode material mass on metal extraction was investigated in 50 mL of 0.4 mol·L^−1^ H_3_PO_4_ at 80 °C, in the presence of 0.2 g Cu and at a stirring speed of 600 rpm for 60 min. The mass of cathode material was varied from 0.2 to 1.0 g. The results are presented in Figure 10.

An overall decreasing trend in leaching efficiency was observed with increasing cathode mass, although the extent of this effect differed among the investigated elements. Lithium extraction remained nearly quantitative at lower solid loadings (≈100% at 0.2 and 0.4 g), followed by a gradual decrease to 94%, 79%, and 75% at 0.6, 0.8, and 1.0 g, respectively. This behavior suggests that Li dissolution is relatively less sensitive to increasing solid concentration compared to transition metals.

In contrast, a more pronounced decline was observed for Ni, Co, and Mn. Nickel extraction decreased from approximately 99% at 0.2–0.4 g to 81%, 59%, and 45% at higher masses. A similar trend was noted for cobalt, with extraction decreasing from about 99.9% and 99.2% at 0.2 and 0.4 g to 76%, 52%, and 40%, respectively. Manganese showed high extraction efficiency at low solid loadings (95–97% at 0.2–0.4 g), followed by a more significant drop to 72%, 48%, and 36% as the cathode mass increased further.

The observed decrease in leaching efficiency may not be attributed solely to the change in the solid-to-liquid ratio. Although increasing the mass of cathode material at constant solution volume reduces the availability of acid per unit mass of solid, the magnitude of the decline, particularly for transition metals, suggests that additional factors could be involved.

One possible explanation is related to the fixed amount of metallic copper used as a reducing agent. As the cathode mass increases, the relative amount of reducible species (such as Co^3+^ and Mn^4+^) also increases, while the electron-donating capacity of copper remains constant. This may lead to a gradual decrease in the effective reducing power per unit mass of solid, potentially resulting in incomplete reduction and lower dissolution efficiencies, especially for redox-sensitive elements.

This interpretation is consistent with the more pronounced decrease observed for Co and Mn, whose dissolution is strongly dependent on reduction to lower oxidation states. However, it should be noted that other factors may also contribute to the observed behavior. For example, higher solid loadings could promote the formation of surface layers, such as copper phosphate phases, which may partially hinder further leaching. In addition, increased particle concentration may introduce local mass transfer limitations or affect the dispersion of solid particles in the solution.

A similar dependence of extraction efficiency on solid loading has been reported in Cu-assisted leaching systems, where a fixed reductant dosage may become insufficient at higher solid concentrations. Such behavior was also observed in our previous study on the LiCoO_2_ system [35], where a decrease in Co extraction was noted with increasing cathode mass at a constant copper dosage. In the present multi-component LiNi_0.65_Co_0.25_Mn_0.1_O_2_ system, this effect may be even more pronounced due to the simultaneous presence of multiple redox-active metals.

Considering both extraction efficiency and reagent utilization, a cathode material mass of 0.4 g per 50 mL solution was selected as a suitable operating condition for subsequent experiments.

#### 2.3.4. Effect of Stirring Speed on the Leaching Efficiencies of Li, Ni, Co, and Mn

**Figure 11:**
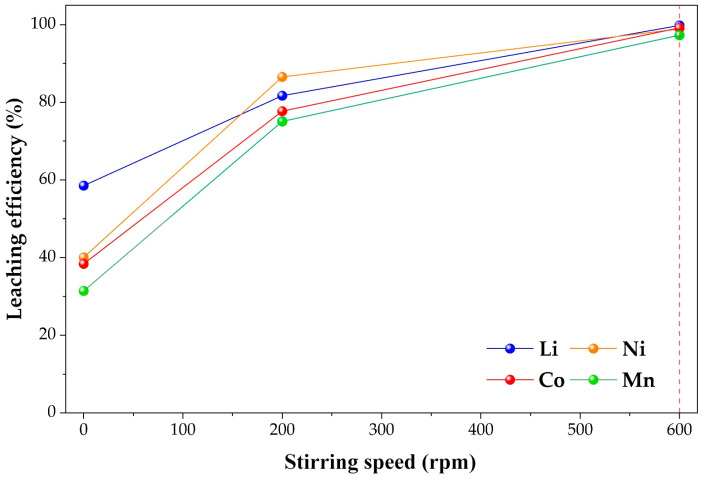
Effect of stirring speed on the leaching efficiencies of Li, Ni, Co, and Mn (0.4 mol·L^−1^ H_3_PO_4_, 0.2 g Cu, 80 °C, 60 min). The dashed line indicates the optimal stirring speed (600 rpm).

The influence of stirring speed on metal extraction was evaluated in 50 mL of 0.4 mol·L^−1^ H_3_PO_4_ at 80 °C, using 0.4 g of cathode material and 0.2 g Cu for 60 min. The stirring rate was varied between static conditions (0 rpm), 200 rpm, and 600 rpm. The results are shown in Figure 11.

A significant enhancement in metal extraction was observed with increasing agitation intensity. Under static conditions, dissolution efficiencies were relatively low, with lithium, nickel, cobalt, and manganese reaching approximately 59%, 40%, 38%, and 31%, respectively, indicating that mass transfer limitations may restrict the overall process in the absence of stirring.

Introducing moderate stirring at 200 rpm led to a substantial increase in metal recovery, with lithium, nickel, cobalt, and manganese reaching approximately 82%, 87%, 78%, and 75%, respectively. This improvement may be attributed to enhanced mixing and a reduction in the thickness of the liquid boundary layer, which facilitates the transport of reactive species between the solid surface and the bulk solution.

Further increasing the stirring speed to 600 rpm resulted in near-complete extraction of all investigated metals, with lithium, nickel, cobalt, and manganese reaching approximately 100%, 99%, 99%, and 97%, respectively. The pronounced increase between 0 and 200 rpm, followed by a smaller improvement between 200 and 600 rpm, may indicate a gradual reduction in external mass transfer limitations with increasing agitation intensity.

These observations are consistent with literature reports, where increased stirring reduces the thickness of the liquid boundary layer and enhances mass transfer across the solid–liquid interface. Similar behavior has been reported for LiNi_0.5_Co_0.2_Mn_0.3_O_2_ systems, where metal extraction increases significantly at low to moderate stirring rates, while further increases in agitation have only a limited effect. This suggests that, beyond a certain stirring speed, diffusion resistance in the liquid film surrounding the particles becomes sufficiently low and no longer dominates the overall process. Under such conditions, the leaching behavior may be governed by a combination of reaction and structural factors [32,44].

At higher stirring speeds, improved hydrodynamic conditions likely reduce external diffusion resistance and allow the leaching process to proceed more efficiently. Based on these observations, a stirring speed of 600 rpm was selected as an appropriate operating condition for subsequent experiments.

#### 2.3.5. Effect of Temperature and Time on the Leaching Efficiencies of Li, Ni, Co, and Mn

**Figure 12:**
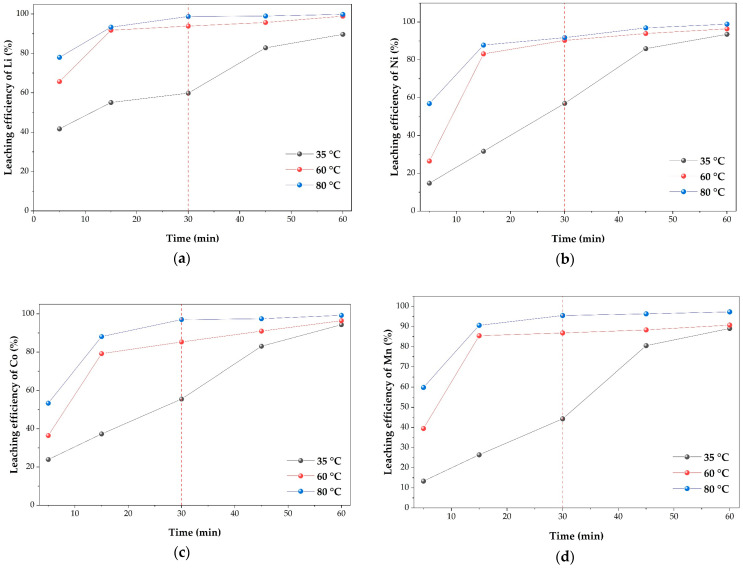
Time-dependent leaching efficiencies of (**a**) Li, (**b**) Ni, (**c**) Co, and (**d**) Mn at different temperatures (0.4 mol·L^−1^ H_3_PO_4_, 0.4 g cathode material, 0.2 g Cu, 600 rpm). The dashed lines indicate the time at which the leaching process approaches completion (30 min).

The effect of temperature on the time-dependent leaching behavior of Li, Ni, Co, and Mn was investigated under optimal conditions (0.4 mol·L^−1^ H_3_PO_4_, 0.4 g cathode material, 0.2 g Cu, 600 rpm) at 35, 60, and 80 °C (Figure 12).

A pronounced temperature-dependent acceleration of metal dissolution was observed for all investigated elements, particularly during the initial stage (0–15 min). Increasing the temperature from 35 to 60 °C significantly enhanced the leaching rates, while at 80 °C most of the extraction occurred within the first 15–30 min.

Lithium exhibited a clear temperature dependence in both dissolution rate and final extraction. At 35 °C, extraction gradually increased to ~90% after 60 min, whereas at 60 and 80 °C, near-complete extraction (>99%) was achieved, with most of the dissolution occurring within the first 15–30 min. Although thermodynamic analysis indicates that lithium dissolution is highly favorable over the entire temperature range (ΔG < −260 kJ·mol^−1^), the observed behavior suggests that lithium release is not thermodynamically limited but rather governed by kinetic factors and the structural stability of the layered material.

Nickel dissolution is thermodynamically favorable at all temperatures (ΔG ≈ −70 to −66 kJ·mol^−1^), indicating that its dissolution is not limited by thermodynamic constraints. However, the strong temperature dependence observed experimentally suggests that nickel release is controlled by kinetic factors and structural constraints within the cathode material.

Cobalt and manganese exhibited a strong dependence on both temperature and time. For both elements, extraction increased rapidly with temperature, particularly during the initial stage, while the differences in final extraction between 60 and 80 °C remained relatively limited. Thermodynamic analysis confirms that cobalt dissolution becomes favorable only in the presence of Cu, whereas manganese reduction to Mn^2+^ is thermodynamically favorable even in acidic conditions (negative ΔG), but Mn^2+^ is stable only under sufficiently reducing conditions. Therefore, the observed temperature dependence indicates that temperature mainly accelerates the kinetics of Cu-mediated redox reactions, stabilizing Mn^2+^ in solution and preventing its reoxidation or precipitation, as well as promoting structural destabilization of the cathode material.

Despite the clear temperature dependence, detailed kinetic modeling was not feasible. The leaching curves are characterized by a very rapid increase in extraction within the first 15–30 min, followed by a plateau, especially at higher temperatures. This results in a limited number of experimental points in the region where the reaction rate is most informative. In addition, the variation in curve shape with temperature suggests that different mechanisms may dominate under different conditions. Consequently, although reasonable fits could be obtained under specific conditions (e.g., at lower temperatures), no single kinetic model provided a consistent and reliable description of the entire dataset, as reflected by low correlation coefficients when all data points were considered.

Thermodynamic analysis further indicates that increasing temperature does not significantly change the driving force for metal dissolution (ΔG remains negative) but primarily accelerates reaction kinetics. At the same time, higher temperatures favor the formation of copper phosphate phases (more negative ΔG), which may influence the concentration of Cu^2+^ in solution and consequently affect the redox equilibrium.

From a practical perspective, most metals reach high extraction levels within 30 min at 80 °C, suggesting that prolonged leaching does not significantly improve recovery. Therefore, 80 °C and 30 min can be considered sufficient operating conditions for efficient metal extraction.

Importantly, the combined kinetic and thermodynamic behavior suggests that the leaching process is likely governed by multiple coupled steps rather than a single dominant rate-controlling mechanism (e.g., diffusion or surface reaction control). The rapid initial extraction followed by a plateau, together with the temperature-dependent changes in curve shape, indicates that several mechanisms contribute to the overall kinetics. This indicates that the rate-controlling step is not constant but evolves during leaching, which explains why no single kinetic model can adequately describe the entire process. In the initial stage, proton-assisted dissolution promotes rapid lithium extraction and partial release of transition metals from the surface of the cathode material. At the same time, metallic copper acts as an electron donor, enabling the reduction of higher-valence species such as Co^3+^ and Mn^4+^ to their soluble divalent forms. As the process progresses, the layered NMC structure can undergo reconstruction into spinel-like intermediate phases, which subsequently dissolve, contributing to the observed decrease in reaction rate. In parallel, copper is oxidized and transformed into copper phosphate phases through precipitation reactions, which may modify both the available copper surface and the local redox conditions. Therefore, the overall leaching kinetics may be interpreted as a superposition of surface reaction, redox-controlled dissolution, structural transformation, and precipitation processes, rather than a single rate-controlling mechanism. This interpretation is further supported by the phase evolution and morphological changes observed in the solid residues, as discussed in the following section.

### 2.4. Structural Evolution of the Leaching Residue

**Figure 13:**
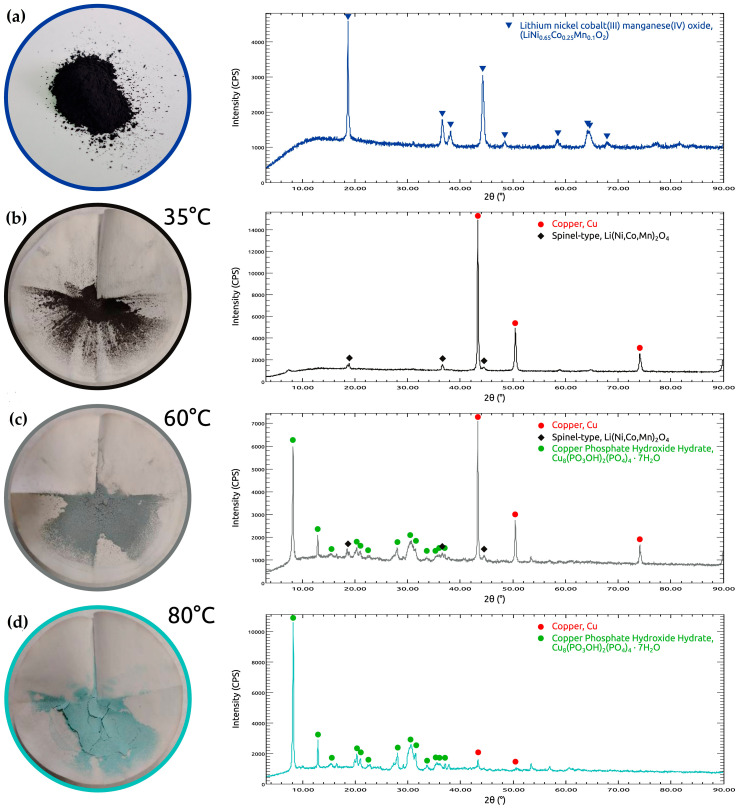
XRD patterns of the pristine cathode material and leaching residues obtained at different temperatures: (**a**) pristine material; (**b**) residue at 35 °C; (**c**) residue at 60 °C; (**d**) residue at 80 °C.

The crystalline structure of the pristine cathode material and the solid residues obtained after leaching at different temperatures were investigated by XRD (Figure 13). The diffraction pattern of the initial sample shows only reflections corresponding to layered LiNi_0.65_Co_0.25_Mn_0.1_O_2_, indicating that the starting material predominantly consists of a layered NCM phase.

After leaching in phosphoric acid in the presence of copper powder, significant changes in phase composition were observed, consistent with the temperature-dependent leaching behavior discussed in the previous section. In the residue obtained at 35 °C, the layered phase is no longer detected, and the solid consists mainly of metallic Cu together with a spinel-type lithium transition-metal oxide belonging to the Li(Ni,Co,Mn)_2_O_4_ structural family. The appearance of this phase suggests that the original layered structure undergoes destabilization and reconstruction during the early stage of leaching, indicating that dissolution may proceed via the formation of an intermediate oxide phase rather than direct decomposition.

At 60 °C, the phase composition becomes more complex. In addition to metallic Cu and the spinel-type Li(Ni,Co,Mn)_2_O_4_ phase, new diffraction peaks assigned to copper phosphate hydroxide hydrate, Cu_8_(PO_3_OH)_2_(PO_4_)_4_·7H_2_O, appear in the residue. This observation suggests that two processes occur in parallel: structural transformation of the cathode material and progressive oxidation of copper, followed by precipitation of copper phosphate phases. This behavior is in agreement with thermodynamic predictions, which indicate that the formation of copper phosphates becomes increasingly favorable with temperature.

At 80 °C, the spinel-type Li(Ni,Co,Mn)_2_O_4_ phase is no longer detected, and the residue is dominated by Cu_8_(PO_3_OH)_2_(PO_4_)_4_·7H_2_O together with residual metallic Cu. This indicates that the reconstructed oxide phase is likely a metastable transient intermediate, which is further dissolved as leaching proceeds under more severe conditions. The persistence of metallic Cu suggests that copper participates progressively in redox reactions rather than being consumed instantaneously.

To further support the structural evolution of the residue, semi-quantitative phase analysis was performed using the reference intensity ratio (RIR) method. The results are summarized in Table 2.

The pristine material consists entirely of the layered LiNi_0.65_Co_0.25_Mn_0.1_O_2_ phase. After leaching at 35 °C, the layered structure is completely decomposed, and the residue is composed predominantly of metallic copper (89%) and a spinel-type phase (11%). At 60 °C, a transition regime is observed, characterized by the formation of copper phosphate hydroxide hydrate (45%), together with metallic copper (47%) and a minor fraction of spinel (8%). At 80 °C, the residue is dominated by copper phosphate (92%), while only a small fraction of metallic copper (8%) remains, and the spinel phase is no longer detected. These results clearly demonstrate a temperature-dependent phase evolution involving the complete breakdown of the layered structure, the formation of a transient spinel-type intermediate, and its subsequent transformation into a stable copper phosphate phase. The reported values should be considered as approximate phase proportions rather than absolute quantitative values, as the RIR method provides semi-quantitative estimates and is associated with inherent uncertainties, particularly in multi-phase systems with overlapping diffraction peaks; however, it reliably captures the relative trends in phase evolution.

Taken together, the qualitative XRD observations and semi-quantitative RIR analysis indicate that the phase evolution follows a temperature-dependent sequence. The disappearance of the layered LiNi_0.65_Co_0.25_Mn_0.1_O_2_ phase at low temperature is consistent with previous reports showing that acid attack on NCM materials typically begins with lithium extraction and destabilization of the layered framework [3,47]. Delithiation of Ni-rich layered oxides is known to induce transition-metal migration and structural rearrangement, frequently leading to the formation of spinel-like structures [48,49,50]. Similar layered-to-spinel transformations have been widely reported in related NCM materials under conditions of delithiation, electrochemical cycling, or thermal degradation [51,52].

At higher temperatures, the disappearance of the intermediate spinel-like phase and the predominance of Cu_8_(PO_3_OH)_2_(PO_4_)_4_·7H_2_O suggest that the reconstructed oxide is not stable under the applied leaching conditions. This behavior is consistent with two-step dissolution mechanisms proposed for NCM materials, in which lithium extraction and structural reconstruction are followed by gradual dissolution of the transformed oxide framework [3,47].

It should be noted that the copper phosphate phase identified by XRD, Cu_8_(PO_3_OH)_2_(PO_4_)_4_·7H_2_O, differs from the simplified phase Cu_3_(PO_4_)_2_ used in the thermodynamic calculations. This difference arises from the fact that thermodynamic modeling typically relies on idealized, anhydrous reference compounds available in the database, whereas the actual precipitated phases in aqueous systems are often hydrated and structurally more complex.

The experimentally observed phase represents a hydrated and protonated form of copper phosphate, formed under specific pH and solution conditions, where phosphate speciation (H_2_PO_4_^−^/HPO_4_^2−^) and water incorporation play a significant role. Therefore, Cu_8_(PO_3_OH)_2_(PO_4_)_4_·7H_2_O can be considered a structurally modified derivative of the thermodynamically predicted copper phosphate phase.

This confirms that the thermodynamic calculations correctly predict the tendency for copper phosphate formation, while the exact phase composition is governed by solution chemistry and crystallization conditions.

**Figure 14:**
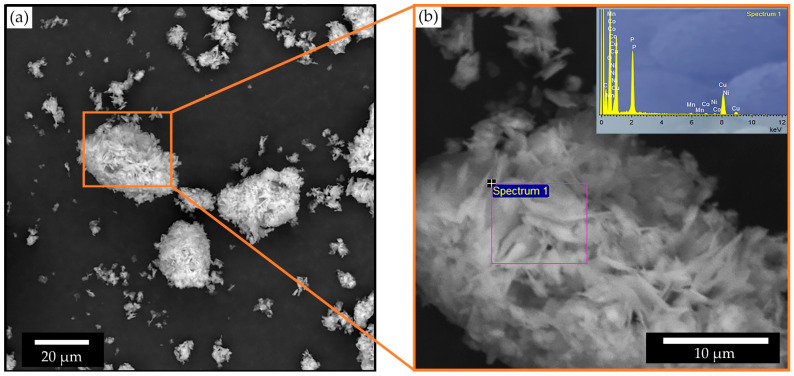
SEM microphotograph of the leaching residue obtained at 80 °C: (**a**) agglomerated particle distribution at lower magnification; (**b**) detailed plate-like morphology of a selected agglomerate with corresponding EDS spectrum.

The chemical composition of the residue obtained at 80 °C was further examined by SEM-EDS analysis (Figure 14). The EDS results show that the solid is mainly composed of O (53.18 at.%), Cu (29.94 at.%), and P (14.63 at.%), while Mn (0.52 at.%), Co (0.96 at.%), and Ni (0.78 at.%) are present only in trace amounts. This confirms that most transition metals were transferred into the leach solution, consistent with the high leaching efficiencies observed experimentally.

SEM observations further support the structural evolution revealed by XRD. The pristine cathode material consists of compact agglomerates of fine primary particles, typical for NCM powders. After leaching at 80 °C, the residue exhibits a strongly altered morphology with irregular agglomerates and fragmented particles, indicating substantial degradation of the original structure. This transformation may be attributed to the dissolution of the transition-metal oxide framework combined with the formation of copper phosphate-rich phases.

Additional insight into the agglomerate size of the leaching residue is provided in Figure S4 (Supplementary Materials), showing that agglomerates are predominantly in the range of approximately 10–80 µm, with noticeable variation in size.

Overall, the combined XRD, SEM, and EDS results suggest that the leaching process proceeds through temperature-dependent structural transformation of the cathode material, coupled with Cu-mediated redox reactions and precipitation of copper phosphate phases, rather than simple direct dissolution of the original layered oxide.

### 2.5. Material Balance and Element Distribution

The distribution of elements between the leach solution and the solid residue was evaluated in order to establish the mass balance of the system under optimized conditions.

As shown in Table 3, the initial masses of Li, Ni, Co, and Mn in the cathode material were 31.2400 mg, 118.1600 mg, 80.1200 mg, and 87.8800 mg, respectively. After leaching in 50 mL of solution, most of the metals were transferred into the liquid phase, with recoveries exceeding 97% for all elements.

Only trace amounts of metals remained in the solid phase, indicating that the cathode material was nearly completely dissolved. The total mass of the leach residue was approximately 0.4412 g. The copper concentration in the leach solution was 19.16 mg·L^−1^, corresponding to approximately 0.96 mg of Cu, which indicates that more than 99% of copper remained in the solid phase.

In agreement with the phase composition discussed in the previous section, the solid residue is predominantly composed of the copper phosphate phase Cu_8_(PO_3_OH)_2_(PO_4_)_4_·7H_2_O, with only a very small fraction of residual metallic copper. The negligible amounts of Ni, Co, and Mn in the residue further confirm that the solid phase is not dominated by undissolved cathode material, but rather by the formation of this secondary copper phosphate phase during the leaching process.

Overall, the leaching process is governed by a coupled dissolution-reduction-precipitation mechanism, in which metallic copper enables the reduction of transition metals while simultaneously undergoing oxidation and subsequent precipitation as copper phosphate.

From a process perspective, the formation of copper phosphate does not represent an irreversible sink for copper, but rather an intermediate phase that can be further processed. Thermodynamic analysis of the Cu-H_3_PO_4_-H_2_O system indicates that copper phosphate phases are in equilibrium with dissolved copper species. Under acidic conditions and in the presence of phosphate ligands, the solubility of copper is enhanced due to the formation of aqueous copper-phosphate complexes, shifting the equilibrium toward dissolved species and enabling the re-dissolution of copper [45]. Furthermore, it has been demonstrated that phosphate ligands form stable complexes with Cu(II), reducing the concentration of free Cu^2+^ in solution and thus limiting its availability for electrochemical reduction, although electrodeposition can still proceed via the remaining free Cu^2+^ fraction, reflecting a dynamic equilibrium between complexed and electrochemically active copper species [53].

In addition, copper can be recovered from phosphate-containing electrolytes by electrochemical reduction to metallic Cu, even when present in complexed forms [54]. Although direct experimental regeneration was not performed in this study, the thermodynamic analysis and literature evidence strongly support the feasibility of copper recovery and reuse. These considerations indicate that the copper phosphate residue should be regarded as a temporary storage form of copper rather than a final waste phase, supporting the concept of copper acting as a recyclable reductant within the process. These findings further emphasize the role of copper as an effective reductant with potential for recovery and reuse. To place these results in a broader context, a comparison with previously reported leaching systems is presented in the following section.

### 2.6. Comparison with Conventional Reductive Leaching Systems

The performance of phosphoric acid-based leaching systems for NMC cathodes is strongly influenced by the type of reducing agent, which determines whether the process remains selective or enables complete metal dissolution.

To provide a systematic comparison, representative phosphoric acid-based reductive leaching systems reported in the literature are summarized in Table 4.

As shown in Table 4, systems based on H_3_PO_4_ combined with hydrogen peroxide typically achieve high lithium extraction under mild conditions, while the dissolution of transition metals such as Co, Ni, and Mn remains limited. This selective behavior is associated with the formation of stable phosphate-containing residues, resulting in predominantly lithium recovery rather than complete metal dissolution [36].

Similarly, systems employing oxalic acid as a reductant exhibit selective dissolution behavior, with high nickel extraction but significantly lower recovery of cobalt and manganese. Furthermore, lithium recovery is not integrated within the same process, requiring additional separation steps [37].

In contrast, systems based on citric acid enable simultaneous dissolution of Li, Ni, Co, and Mn with high extraction efficiencies. This behavior is attributed to the combined reduction-complexation mechanism, where citric acid acts both as a reductant and a complexing agent. However, the formation of stable metal–organic complexes increases solution complexity and may complicate downstream processing [44].

Another approach involves reduction roasting with graphite, where cathode materials are converted into more soluble low-valent phases prior to phosphoric acid leaching. Although this method enables near-complete metal extraction, it requires high-temperature calcination (~650 °C) and introduces additional processing steps, increasing overall energy consumption and operational costs [55].

In contrast, the H_3_PO_4_-Cu system developed in this study enables direct reductive leaching under mild conditions, achieving near-complete extraction of Li, Ni, Co, and Mn without the addition of external liquid reductants. Importantly, the copper used in this study was sourced from spent lithium-ion batteries (current collectors), rather than introduced as an external reagent. During the process, copper acts as both an electron donor and a redox mediator and is subsequently transformed into a copper phosphate phase, which can be further processed, with potential for recovery and reuse, as discussed in Section 2.5.

Overall, the comparison highlights that the proposed system combines high extraction efficiency with reduced reagent consumption and simplified process design. In contrast to other approaches, it avoids the use of external liquid reductants, minimizes the formation of complex organic species, and eliminates the need for energy-intensive pretreatment steps. These features contribute to improved process efficiency and support the potential for more sustainable and circular recycling of NMC cathode materials.

## 3. Materials and Methods

### 3.1. Materials and Reagents

A total of 40 spent lithium-ion batteries from different commercial manufacturers were collected for material recovery. Before dismantling, all batteries were fully discharged to ensure safe handling.

The cathode chemistry was verified according to the methodology described by Medić et al. [56], and only cells containing layered LiNiCoMnO_2_ cathodes were selected for further processing.

The batteries were manually dismantled, allowing separation of the metallic casing, electrode assemblies, and polymer separator. The active cathode material was detached from the aluminum current collector by controlled thermal treatment, after which it was collected, homogenized, and stored for subsequent leaching experiments. The recovered aluminum foil remained suitable for direct recycling.

The anode, consisting of copper foil coated with carbonaceous material, was washed and mechanically cleaned to obtain pure copper foil. A portion of the recovered copper was milled into fine powder and used as a reducing agent in the leaching experiments, while the remaining copper was preserved for recycling.

Residual cathode particles retained on the separator were recovered by controlled combustion. Through this procedure, all battery components were either reintegrated into the experimental workflow or directed to recycling streams, ensuring maximal utilization of the materials.

Phosphoric acid (H_3_PO_4_, Zorka Pharma, Šabac, Serbia) was used as the leaching agent. The elemental composition of the recovered LiNiCoMnO_2_ cathode material was determined after complete digestion in aqua regia (HNO_3_:HCl = 1:3, Merck, Darmstadt, Germany). All reagents were of analytical grade, and deionized water was used for the preparation of all solutions.

### 3.2. Analytical Methods

The elemental composition of the recovered LiNiCoMnO_2_ cathode material was determined before leaching, and the concentrations of Li, Ni, Co, and Mn in solution were monitored throughout the experiments using inductively coupled plasma optical emission spectrometry (ICP-OES, Optima 8300, PerkinElmer, Waltham, MA, USA).

The crystalline phases of the pristine material and the solid residues obtained after leaching were identified by X-ray diffraction (XRD, Rigaku MiniFlex 600, Tokyo, Japan) using Cu Kα radiation (λ = 0.154 nm), operated at 40 kV and 15 mA.

In addition to qualitative phase identification, semi-quantitative phase analysis was performed using the Reference Intensity Ratio (RIR) method implemented in PDXL 2 software (Rigaku), based on reference patterns from the ICDD PDF-2 (2026) database. The analysis relies on integrated peak intensities to estimate relative phase abundances. The obtained values should be considered semi-quantitative, as the method does not account for factors such as preferred orientation, crystallite size effects, or microabsorption. The detection limit of the method is approximately 1%.

The surface morphology, microstructural features, and elemental composition of both the pristine cathode material and the solid residues were examined by scanning electron microscopy coupled with energy-dispersive X-ray spectroscopy (SEM-EDS, Tescan VEGA 3 LMU, Brno, Czech Republic), operated at an accelerating voltage of 20 kV. Data acquisition and analysis were performed using INCA software (version 5.05).

Before experimental investigations, a detailed thermodynamic analysis of the system was conducted. Thermodynamic analysis of the investigated leaching system was performed using a combination of HSC Chemistry (version 10.8.1.2) and Hydra/Medusa software (version 2010) in order to evaluate reaction feasibility, phase stability, and species distribution under the defined conditions.

This approach allows prediction of the behavior of the initial material under defined process conditions, identification of key controlling parameters (pH, temperature, redox potential), and optimization of the leaching process. Furthermore, this modeling strategy reduces the number of required experimental trials, leading to savings in reagents, time, and generated waste. The combined use of Gibbs free energy calculations, speciation diagrams, and Pourbaix diagrams enabled a comprehensive understanding of the leaching behavior, including metal dissolution, redox transformations, and precipitation phenomena. This approach enables a systematic understanding of the influence of pH, redox conditions, and solution composition on metal leaching and supports the interpretation of experimental results. It should be noted that the analysis is based on equilibrium conditions and does not account for kinetic limitations.

### 3.3. Leaching Procedure

Leaching studies were conducted in a 250 mL double-walled glass reactor equipped with a condenser to minimize solution loss and a thermometer for continuous temperature monitoring. The reactor was mounted on a hot plate with magnetic stirring. The total volume of leaching solution in each experiment was fixed at 50 mL.

The experimental design comprised systematic variation in the following parameters:

- Presence of reducing agent—experiments were performed both without copper and with 0.2 g of copper powder added as a reducing agent;
- Phosphoric acid concentration (0.2, 0.4, 0.6, and 0.8 mol·L^−1^);
- Solid-to-liquid ratio, controlled by varying the mass of cathode material (0.2, 0.4, 0.6, 0.8, and 1.0 g) while maintaining a constant copper dosage of 0.2 g when applicable;
- Stirring rate (0, 200, and 600 rpm);
- Reaction temperature (35, 60, and 80 °C).

Unless otherwise specified, 0.2 g of copper powder was introduced as the reducing agent.

During each experiment, liquid samples (1.0 mL) were withdrawn at predetermined time intervals (5, 15, 30, 45, and 60 min). The aliquots were filtered, diluted to a defined volume, and analyzed for metal content.

Upon completion of leaching, solid residues were separated by filtration, thoroughly washed with distilled water, and dried. To elucidate structural and morphological changes induced by leaching, representative residues were characterized by XRD and SEM-EDS analysis.

## 4. Conclusions

This study demonstrates that copper-assisted leaching in phosphoric acid provides an effective and chemically simplified approach for the recovery of Li, Ni, Co, and Mn from LiNi_0.65_Co_0.25_Mn_0.1_O_2_ cathode materials. The results show that metallic copper derived from spent battery components acts as both a reducing agent and a redox mediator, enabling efficient dissolution of transition metals, particularly Co and Mn, which are otherwise thermodynamically unfavorable under non-reducing conditions.

Process optimization revealed that 0.4 mol·L^−1^ H_3_PO_4_, a stirring speed of 600 rpm, and a temperature of 80 °C provide near-complete extraction of all investigated metals within 30 min. The leaching process could not be described by a single kinetic model, indicating that it is governed by multiple coupled steps involving surface reactions, redox processes, and structural transformations of the cathode material.

Thermodynamic analysis confirmed that acidic and reducing conditions (pH 1–3, Eh 0–0.5 V) represent the optimal domain for metal dissolution, where Li^+^, Ni^2+^, Co^2+^, and Mn^2+^ are stable in solution. In this region, copper undergoes progressive transformation from a metallic reductant to dissolved Cu^2+^ and ultimately to stable phosphate phases, in agreement with calculated ΔG values and speciation analysis.

Structural characterization confirmed a temperature-dependent phase evolution from the layered NCM structure to spinel-like intermediates, followed by their gradual dissolution and the formation of copper phosphate phases. The formation of Cu_8_(PO_3_OH)_2_(PO_4_)_4_·7H_2_O was found to be thermodynamically favored at elevated temperatures and represents the dominant phase in the final residue.

Mass balance analysis confirmed that more than 97% of all target metals were transferred into the leach solution, while copper remained predominantly in the solid phase, supporting its role as a recyclable reducing agent. This interpretation is further supported by thermodynamic considerations and literature data indicating that copper bound in phosphate phases can be re-dissolved under acidic conditions and subsequently recovered as metallic copper by electrochemical reduction, as supported by thermodynamic analysis and literature evidence, enabling its reintegration into the process.

Overall, the proposed H_3_PO_4_-Cu system offers a promising alternative to conventional hydrometallurgical routes by eliminating the need for external homogeneous reducing agents such as hydrogen peroxide. The combination of high efficiency, simplified chemistry, and potential for reagent reuse highlights its applicability for sustainable recycling of NMC cathode materials. Further studies should focus on detailed kinetic analysis and optimization of copper utilization in the leaching system.

## Figures and Tables

**Table 1:** Calculated thermodynamic parameters (ΔH^θ^, ΔS^θ^, ΔG^θ^, and log K) for reactions of leaching process (graphical representation shown in Supplementary Materials Figure S3).

T, °C	Parameters ^1^	Equation (1)	Equation (2)	Equation (3)	Equation (4)	Equation (5)	Equation (6)	Equation (7)	Equation (8)	Equation (9)	Equation (10)
0	H^θ^ (kJ)	−254.37	−106.19	−118.77	−357.26	−285.02	−219.50	69.69	−19.79	10.18	21.86
	ΔS^θ^ (J/K)	22.55	−118.54	−623.72	−372.47	−164.28	−162.53	853.15	−4.63	−102.83	−161.97
	ΔG^θ^ (kJ/mol^−1^)	−260.53	−73.82	51.60	−255.52	−240.15	−175.11	−163.35	−18.52	38.27	66.10
	log K	49.82	14.12	−9.87	48.87	1.24	33.49	31.24	3.54	−7.31	−12.64
35	H^θ^ (kJ)	−243.46	−101.2	−99.02	48.87	−271.66	−206.77	122.56	−24.92	1.56	12.34
	ΔS^θ^ (J/K)	61.42	−100.2	−550.81	−285.62	−115.69	−116.19	1036.33	−22.37	−132.70	−194.92
	ΔG^θ^ (kJ/mol^−1^)	−262.39	−70.34	70.71	−245.46	−236.01	−170.96	−196.79	−18.03	42.45	72.41
	log K	44.48	11.92	−11.99	41.61	1.22	28.98	33.36	3.05	−7.19	−12.27
60	H^θ^ (kJ)	−240.11	−101.38	−99.76	−331.62	−270.31	−205.35	151.37	−42.33	−3.15	7.14
	ΔS^θ^ (J/K)	71.89	−100.69	−553.11	−279.84	−111.48	−111.77	1126.26	−77.31	−147.4	−211.16
	ΔG^θ^ (kJ/mol^−1^)	−264.06	−67.83	84.51	−238.39	−233.17	−168.11	−223.85	−16.57	45.95	77.49
	log K	41.40	10.64	−13.25	37.38	1.21	26.36	35.100	2.59	−7.20	−12.15
80	H^θ^ (kJ)	−237.58	−101.54	−100.60	−330.37	−269.31	−204.34	174.05	−45.92	−6.81	3.09
	ΔS^θ^ (J/K)	79.26	−101.15	−555.57	−276.17	−108.59	−108.83	1192.38	−87.78	−158.09	−222.96
	ΔG^θ^ (kJ/mol^−1^)	−265.57	−65.817	95.59	−232.83	−230.97	−165.91	−247.03	−14.92	49.01	81.83
	log K	39.28	9.736	−14.11	34.42	1.19	24.54	36.54	2.20	−7.25	−12.10
100	H^θ^ (kJ)	−235.22	−101.87	−102.20	−329.76	−268.49	−203.66	197.95	−49.75	−10.61	−1.10
	ΔS^θ^ (J/K)	85.74	−102.06	−559.96	−274.49	−106.33	−106.96	1258.18	−98.34	−168.55	−234.53
	ΔG^θ^ (kJ/mol^−1^)	−267.22	−63.78	106.74	−227.33	−228.82	−163.75	−271.54	−13.06	52.28	86.41
	log K	37.41	8.93	−14.94	31.826	1.187	22.925	38.01	1.83	−7.31	−12.09

^1^ ΔH^θ^—enthalpy change; ΔS^θ^—entropy change; ΔG^θ^—Gibbs free energy change; log K—equilibrium constant.

**Table 2:** Semi-quantitative phase composition of leaching residues estimated using the RIR method based on XRD peak intensities.

Mineral/ Phase Name	Chemical Formula	Pristine (%)	35 °C (%)	60 °C (%)	80 °C (%)
Layered NCM	LiNi_0.65_Co_0.25_Mn_0.1_O_2_	100	n.d.	n.d.	n.d.
Spinel-type	Li(Ni,Co,Mn)_2_O_4_	n.d.	11	8	n.d.
Metallic copper	Cu	n.d.	89	47	8
Copper phosphate hydrate	Cu_8_(PO_3_OH)_2_(PO_4_)_4_·7H_2_O	n.d.	n.d.	45	92

n.d.—not detected.

**Table 3:** Elemental mass balance and distribution between leach solution and solid residue under optimized conditions.

Stream/Parameter	Total Mass (g)	Li (wt%)	Li Mass (mg)	Ni (wt%)	Ni Mass (mg)	Co (wt%)	Co Mass (mg)	Mn (wt%)	Mn Mass (mg)	Note
Feed (cathode material)	0.4000	7.81	31.2400	29.54	118.1600	20.03	80.1200	21.97	87.8800	Target metals in the sample
Cu additive	0.2000	—	—	—	—	—	—	—	—	
Total input (feed + additive)	0.6000	—	31.2400	—	118.1600	—	80.1200	—	87.8800	
Leach solution (50 mL)		—	31.1775	—	116.8602		79.4790		85.4984	Concentration: Li 623.55 mg·L^−1^; Ni 2337.20 mg·L^−1^; Co 1589.58 mg·L^−1^; Mn 1709.97 mg·L^−1^
Leach residue (dry)	0.4412	—	0.0625	—	1.2998		0.6411		2.3815	
Extraction (%)	—	99.80	—	98.90	—	99.20		97.29		

**Table 4:** Comparison of representative phosphoric acid-based reductive leaching systems for LIB cathodes.

System	Reducing Agent	Leaching Conditions	Metal Extraction (%)	Process Features	Environmental/Economic Aspects	Ref.
H_3_PO_4_ + H_2_O_2_	H_2_O_2_ (liquid)	0.8 mol·L^−1^ H_3_PO_4_, 60 °C, 60 min	Li = 98.94%, Ni < 10%, Co < 10%, Mn < 10%	Selective Li leaching; transition metals largely retained in phosphate-containing residue	Requires external H_2_O_2_; reagent consumption and decomposition; additional steps needed for transition metal recovery	[36]
H_3_PO_4_ + H_2_C_2_O_4_	H_2_C_2_O_4_ (organic)	0.65 mol·L^−1^ H_3_PO_4,_ 60 °C, 90 min	Ni = 99.90%, Co = 43.65% Mn = 3.29%,	Selective dissolution of Ni with partial extraction of Co and minimal Mn recovery	Organic acid system reduces reliance on toxic reductants; however, it requires additional reagents and downstream separation steps	[37]
H_3_PO_4_ + C_6_H_8_O_7_	C_6_H_8_O_7_ (organic)	0.2 mol·L^−1^ H_3_PO_4,_ 90 °C, 30 min	Li = 100%, Ni = 93.38%, Co = 91.63%, Mn = 92.00%,	Non-selective leaching with high recovery of all metals due to a combined reduction–complexation mechanism	Green leaching system with low cost and no external inorganic reductant, but generates complex organic leachates that complicate downstream separation	[44]
H_3_PO_4_ + graphite	Graphite (solid, battery-derived)	1 mol·L^−1^ H_3_PO_4,_ 80 °C, 60 min	Li = 99.90%, Ni = 99.80%, Co = 99.70%, Mn = 99.70%	High-efficiency leaching enabled by reduction roasting with graphite, converting high-valence metals to divalent oxides before phosphoric acid leaching	Utilizes waste graphite as a low-cost reductant and avoids chemical reducing agents, but requires high-temperature calcination (650 °C), increasing energy consumption	[55]
H_3_PO_4_ + Cu	Cu (solid, battery-derived)	0.4 mol·L^−1^ H_3_PO_4,_ 80 °C, 60 min	Li = 99.99%, Ni = 99.00%, Co = 99.00%, Mn = 97.00%,	Direct reductive leaching enabled by metallic Cu acting as both electron donor and redox mediator, coupled with simultaneous copper phosphate formation	No external reductants required; reduced reagent consumption; Cu retained in the solid phase with potential for recovery and reuse	[This work]

## Data Availability

The original contributions presented in the study are included in the article. Further inquiries can be directed to the corresponding author.

## Supplementary Materials

The following supporting information can be downloaded at: https://www.mdpi.com/article/10.3390/molecules31091502/s1, Figure S1: SEM microphotograph of the cathode material used for particle size analysis, with particle boundaries defined in ImageJ (yellow overlay) for Feret diameter measurement; Figure S2: SEM microphotograph of the cathode material used for agglomerate size estimation, showing defined agglomerate regions used for size evaluation; Figure S3: Calculated thermodynamic parameters for reactions: (a) ΔH^θ^, (b) ΔS^θ^, and (c) ΔG^θ^ and (d) log K for reactions of leaching process; Figure S4: SEM microphotograph of leaching residue used for agglomerate size estimation, showing defined agglomerate regions used for size evaluation.

## Abbreviations

The following abbreviations are used in this manuscript:

CRMs	Critical raw materials
EU	European Union
LIBs	Lithium-ion batteries
LCO	LiCoO_2_
NMC	LiNi_x_Co_y_Mn_1−x−y_O_2_
LMO	LiMn_2_O_4_
LFP	LiFePO_4_
DES	Deep eutectic solvents
ICP-OES	Inductively Coupled Plasma Optical Emission Spectrometry
XRD	X-ray Diffraction
SEM-EDS	Scanning Electron Microscopy coupled with Energy-Dispersive X-ray Spectroscopy
