# Supplementary material for: Copper-Mediated Leaching of LiNi0.65Co0.25Mn0.10O2 in H3PO4: Thermodynamics, Structural Evolution, and Redox Mechanism
Source: Molecules. 2026 Apr 30;31(9):1502. doi: 10.3390/molecules31091502 (PMC13165113; doi:10.3390/molecules31091502)
Supplement: Supplementary file 1 [file molecules-31-01502-s001.zip › molecules-4275930-supplementary.pdf]

molecules

Article

Copper-Mediated Leaching of $LiNi_{0.65}Co_{0.25}Mn_{0.10}O_2$ in H_3PO_4: Thermodynamics, Structural Evolution, and Redox Mechanism

Ivan Đorđević [1], Dragana Medić [2,*], Nataša Gajić [3], Maja Nujkić [2], Vladan Nedelkovski [2], Sonja Stanković [2] and Aleksandar Cvetković [2]

1 Elixir Prahovo Co., Ltd., Braće Jugovića br. 2, 19330 Prahovo, Serbia; ivan.djordjevic@elixirprahovo.rs
2 Technical Faculty in Bor, University of Belgrade, 19210 Bor, Serbia; mnujkic@tfbor.bg.ac.rs (M.N.); vnedelkovski@tfbor.bg.ac.rs (V.N.); sstankovic@tfbor.bg.ac.rs (S.S.); acvetkovic@tfbor.bg.ac.rs (A.C.)
3 Innovation Center of the Faculty of Technology and Metallurgy, Belgrade Ltd., University of Belgrade, Karnegijeva 4, 11000 Belgrade, Serbia; ngajic@tmf.bg.ac.rs
* Correspondence: dmedic@tfbor.bg.ac.rs; Tel.: +381-604231087

List of Figures

Figure S1. SEM microphotograph of the cathode material used for particle size analysis, with particle boundaries defined in ImageJ (yellow overlay) for Feret diameter measurement.
Figure S2. SEM microphotograph of the cathode material used for agglomerate size estimation, showing defined agglomerate regions used for size evaluation.
Figure S3. Calculated thermodynamic parameters for reactions: (a) ΔH^θ, (b) ΔS^θ, and (c) ΔG^θ and (d) log K for reactions of leaching process
Figure S4. SEM microphotograph of leaching residue used for agglomerate size estimation, showing defined agglomerate regions used for size evaluation.

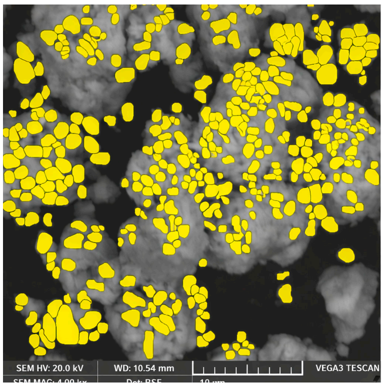

Figure S1. SEM microphotograph of the cathode material used for particle size analysis, with particle boundaries defined in ImageJ (yellow overlay) for Feret diameter measurement.

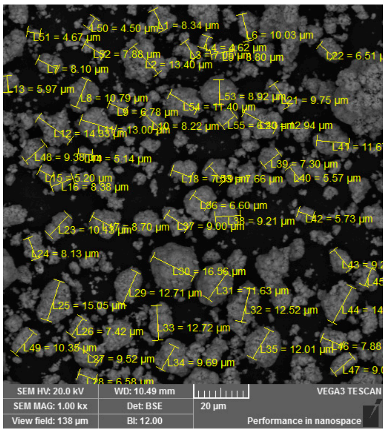

Figure S2. SEM microphotograph of the cathode material used for agglomerate size estimation, showing defined agglomerate regions used for size evaluation.

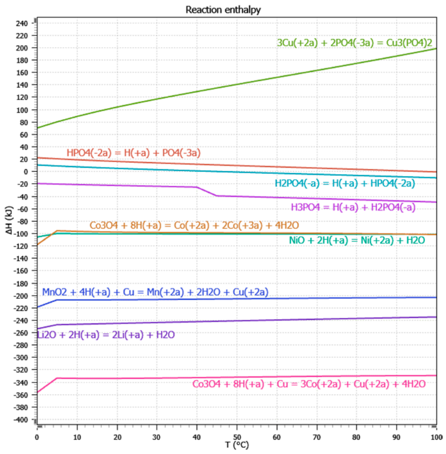

(a)

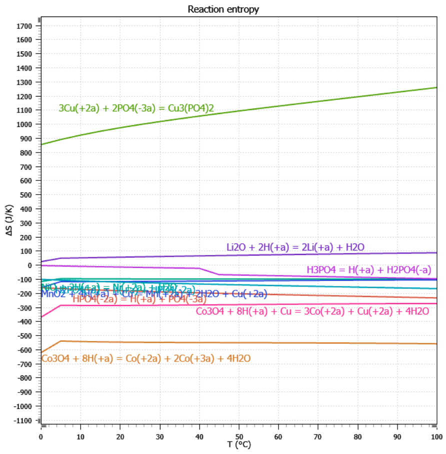

(b)

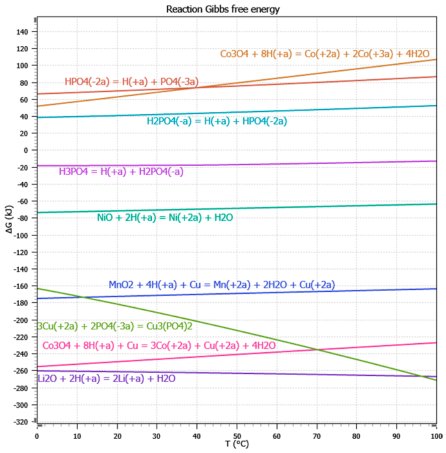

(c)

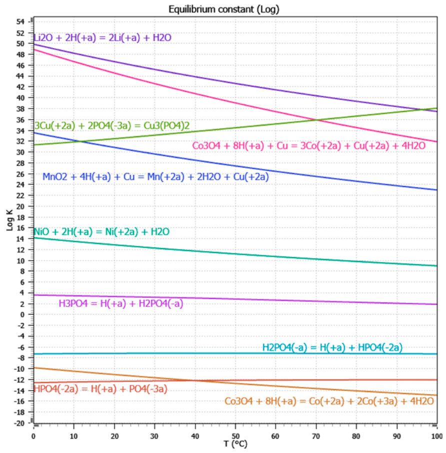

(d)

Figure S3. Calculated thermodynamic parameters for reactions: (a) ΔH^{θ}, (b) ΔS^{θ}, and (c) ΔG^{θ} and (d) log K for reactions of leaching process.

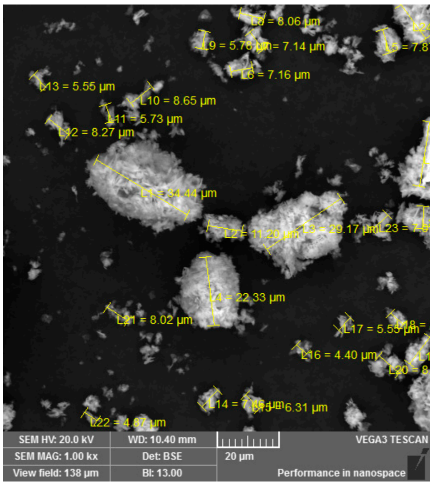

Figure S4. SEM microphotograph of leaching residue used for agglomerate size estimation, showing defined agglomerate regions used for size evaluation.
